# Combined Analysis of Myocardial Deformation and Oxygenation Detects Inducible Ischemia Unmasked by Breathing Maneuvers in Chronic Coronary Syndrome

**DOI:** 10.3389/fcvm.2022.800720

**Published:** 2022-02-24

**Authors:** Barbara Spicher, Kady Fischer, Zoe A. Zimmerli, Kyohei Yamaji, Yasushi Ueki, Carina N. Bertschinger, Bernd Jung, Tatsuhiko Otsuka, Marius R. Bigler, Christoph Gräni, Hendrik von Tengg-Kobligk, Lorenz Räber, Balthasar Eberle, Dominik P. Guensch

**Affiliations:** ^1^Department of Anaesthesiology and Pain Medicine, Inselspital, University Hospital Bern, University of Bern, Bern, Switzerland; ^2^Department of Cardiology, Inselspital, University Hospital Bern, University of Bern, Bern, Switzerland; ^3^Department of Diagnostic, Interventional and Paediatric Radiology, Inselspital, University Hospital Bern, University of Bern, Bern, Switzerland

**Keywords:** coronary artery disease, oxygenation-sensitive imaging, cardiovascular magnetic resonance, feature tracking (CMR-FT), strain, breathing maneuver

## Abstract

**Introduction:**

In patients with chronic coronary syndromes, hyperventilation followed by apnea has been shown to unmask myocardium susceptible to inducible deoxygenation. The aim of this study was to assess whether such a provoked response is co-localized with myocardial dysfunction.

**Methods:**

A group of twenty-six CAD patients with a defined stenosis (quantitative coronary angiography > 50%) underwent a cardiovascular magnetic resonance (CMR) exam prior to revascularization. Healthy volunteers older than 50 years served as controls (*n* = 12). Participants hyperventilated for 60s followed by brief apnea. Oxygenation-sensitive images were analyzed for changes in myocardial oxygenation and strain.

**Results:**

In healthy subjects, hyperventilation resulted in global myocardial deoxygenation (-10.2 ± 8.2%, *p* < 0.001) and augmented peak circumferential systolic strain (-3.3 ± 1.6%, *p* < 0.001). At the end of apnea, myocardial signal intensity had increased (+9.1 ± 5.3%, *p* < 0.001) and strain had normalized to baseline. CAD patients had a similar global oxygenation response to hyperventilation (−5.8 ± 9.6%, *p* = 0.085) but showed no change in peak strain from their resting state (-1.3 ± 1.6%), which was significantly attenuated in comparison the strain response observed in controls (*p* = 0.008). With apnea, the CAD patients showed an attenuated global oxygenation response to apnea compared to controls (+2.7 ± 6.2%, *p* < 0.001). This was accompanied by a significant depression of peak strain (3.0 ± 1.7%, *p* < 0.001), which also differed from the control response (*p* = 0.025). Regional analysis demonstrated that post-stenotic myocardium was most susceptible to de-oxygenation and systolic strain abnormalities during respiratory maneuvers. CMR measures at rest were unable to discriminate post-stenotic territory (*p* > 0.05), yet this was significant for both myocardial oxygenation [area under the curve (AUC): 0.88, *p* > 0.001] and peak strain (AUC: 0.73, *p* = 0.023) measured with apnea. A combined analysis of myocardial oxygenation and peak strain resulted in an incrementally higher AUC of 0.91, *p* < 0.001 than strain alone.

**Conclusion:**

In myocardium of patients with chronic coronary syndromes and primarily intermediate coronary stenoses, cine oxygenation-sensitive CMR can identify an impaired vascular and functional response to a vasoactive breathing maneuver stimulus indicative of inducible ischemia.

## Introduction

Coronary artery disease (CAD) can be classified into subcategories of acute coronary syndromes (ACS) and chronic coronary syndromes (CCS) (1). CCS encompasses a range of clinical scenarios and can include but are not limited to patients who have stable symptoms following revascularization and patients where an obstructive coronary stenosis was observed during screening. In these patients, symptoms and myocardial abnormalities may not be apparent during resting conditions as there may not be a mismatch of oxygen supply to demand. This may rapidly change when the heart is exposed to stressors or vasoactive stimuli that tips this oxygen supply-demand balance. This can induce a cascade of changes in the myocardium including a reduction in tissue perfusion and oxygenation balance, and subsequent diastolic and systolic dysfunction (2, 3). Multiple non-invasive imaging techniques can assess patients at various stages in this cascade (4), and with new developments in cardiovascular magnetic resonance (CMR) there is potential to investigate the triggered change in both myocardial tissue and functional measures associated with inducible ischemia in CCS.

### Oxygenation-Sensitive Cardiovascular Magnetic Resonance

Non-invasive imaging of myocardial ischemia is a rapidly developing field, with a significant expansion in the variety of imaging techniques available to investigate changes in the myocardial tissue perfusion and oxygenation. A concomitant deoxygenation response has been demonstrated using Oxygenation-Sensitive (OS)-CMR (5). OS-CMR does not rely on gadolinium contrast but uses deoxyhemoglobin as an endogenous contrast agent, based on its paramagnetic molecular properties (6). The resulting local magnetic field inhomogeneities cause a loss of regional signal intensity (SI) in CMR images acquired using OS sequences (7–10). In contrast, oxygenated hemoglobin is diamagnetic and leads to weak field stabilization, which does not change SI. Thus, OS-CMR offers an attractive option for non-invasive detection and localization of regional myocardial deoxygenation without the use of exogenous contrast. While OS-CMR can indicate oxygen supply-and-demand mismatch, inducible ischemia is the sequela. Therefore, an additional measure of ventricular dysfunction would be beneficial.

### Assessing Regional Myocardial Function With CMR Feature Tracking

Regional analysis of myocardial strain using CMR feature tracking (FT) is a more recent technique to assess myocardial function. Myocardial strain provides insight into contractile and lusitropic function in which feature tracking techniques follow the relative movement of unique features in the image throughout the cardiac cycle in the longitudinal, circumferential and radial axis. Post-processing software allows for quantification of multiple systolic and diastolic deformation parameters, such as peak systolic strain (PS), time to PS (TTP), and myocardial diastolic strain rate (dSR). The association of changes of OS-CMR-derived myocardial oxygenation with regional myocardial function has not been studied so far in cardiovascular patients. In swine, this combined analysis was performed and FT measurements from OS images were shown to be linked with myocardial deoxygenation at low perfusion pressures (11). With the use of FT software, the OS cines used for oxygenation analysis can be simultaneously interrogated to assess strain parameters.

### Breathing Maneuvers for Provocation of an Endogenous Coronary Vasomotor Response

Pharmacological agents such as adenosine or regadenoson are often used diagnostically to test coronary vasoreactivity. Yet the heart has natural feedback loops to respond to non-pharmacological stimuli as well as exercise, sympathetic function testing (i.e., cold pressor test), and changes in breathing patterns. The mechanisms of hyperventilation and apnea on myocardial oxygenation balance are not fully understood, but a key regulatory pathway appears to be through local blood carbon dioxide (CO_2_) partial pressures. Hyperventilation induces hypocapnia, which is known to be a potent coronary vasoconstrictor. Apnea has an opposing effect increasing CO_2_ and this subsequent hypercapnia induces significant vasodilation in healthy coronary vessels (12, 13). This has been demonstrated using inhaled gas mixtures (14, 15) and paced intentional breathing maneuvers (12, 16). Hypercapnic coronary vasodilation has been described since 1970, and it has been hypothesized that hypercapnia could induce inter-coronary flow redistributions that may result in a steal phenomenon in CAD patients (13). Meanwhile, this assumption has been verified by Fischer et al. (12) using OS-CMR in an animal model of acute coronary stenosis. However, the effect on myocardial contractile function is unknown.

This study aimed to investigate the association of provoked dynamic myocardial oxygenation changes, as measured using OS-CMR, with regional myocardial strain in healthy subjects and patients with well-defined CCS.

## Materials and Methods

### Study Population

The study protocol was approved by the ethics board of the Canton of Bern and complies with the ethical guidelines of the 1975 Declaration of Helsinki. A total of twenty-six patients with a diagnosis of CAD and twelve healthy volunteers in a comparable age-range (50–70 years) were included. Seventeen (45%) of the participants had been included in previous publications (17, 18). All participants had given their written informed consent prior to enrolment into the study. The patients were recruited for their CMR in the time interval between their initial coronary computed tomography or invasive coronary angiography visit (>3 weeks) and their subsequent admission for revascularization. From these diagnostic exams the presence of obstructive CAD was verified and patients were included if at least one untreated major epicardial coronary artery with more than 50% stenosis by quantitative coronary angiography (QCA) was present during the CMR scan, together with at least one patent epicardial vessel. Exclusion criteria included general contraindications to CMR, pregnancy, pre-existing coronary bypass grafts, severe pulmonary disease, and any unstable medical condition. Moreover, patients with a ST-elevation myocardial infarction (STEMI) as reason for the initial angiography were not included in this analysis. Healthy subjects were required to be between the ages of 50–70 years, be non-smokers for the past 6 months and to be without a history of cardiopulmonary disease or pertinent medication. Participants were also asked to abstain from caffeine-rich intake for twelve hours prior to the CMR exam.

### CMR Protocol

**Figure 1:**
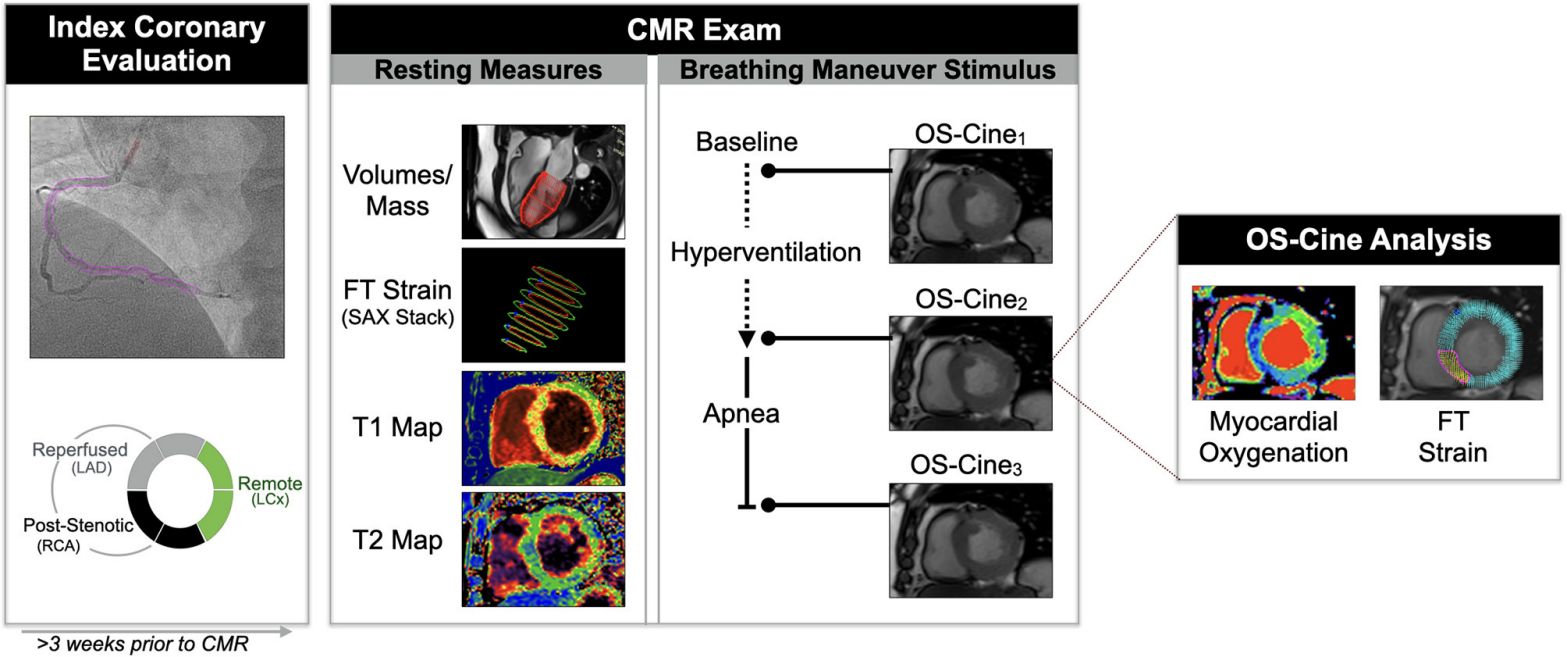
Study methods. From the angiography images, quantitative coronary angiography was used to calculate the diameters stenosis of all coronary territories and the myocardial segments were categorized as post-stenotic, reperfused or remote territory based on the individual coronary anatomy. A contrast-free CMR exam was performed at least 3 weeks after the index angiography. Common functional and tissue characterization sequences were acquired at rest, and an oxygenation-sensitive (OS)-cine was acquired repetitively during a breathing maneuver stimulus. From the OS-cine both signal intensity changes indicating the myocardial oxygenation response and feature tracking (FT) strain parameters were measured for the global myocardium, and per myocardial segment.

All participants underwent a contrast-free exam in a 3T MRI scanner (MAGNETOM Skyra™ or Prisma™, Siemens Healthineers, Erlangen, Germany). A short-axis stack along with two long-axis images were obtained for the analysis of baseline ventricular function parameters. Additionally, native T1 and T2 maps were acquired in a basal and mid-ventricular slice. OS-CMR cines were obtained in these same slice positions. Under resting conditions, a baseline OS cine was acquired during a brief (5–8 s) breath-hold. Participants were then instructed to hyperventilate for 60 s (30 breaths/min paced by a metronome), and immediately following hyperventilation, to maintain apnea at a comfortable exhalation level. Throughout the entire duration of this apneic period, OS-cines were acquired continuously until participants indicated their need to resume breathing (Figure 1).

### CMR Sequence Parameters

All images were obtained at an end-expiratory breath-hold. OS-CMR images were obtained with an ECG-triggered balanced steady-state free precession sequence (TR/TE 3.4/1.70 ms, temporal resolution 40.7 ms, flip angle 35°, voxel size 2.0 x 2.0 x 10.0 mm, matrix 192 x 120, bandwidth 1302 Hz/Px). Standard cine images were acquired with a standard gated balanced steady-state free precession cine sequence (TR/TE 3.3/1.43 ms, 25 cardiac phases, flip angle 65°, voxel size 1.6 x 1.6 x 6.0 mm, matrix 192 x 120, bandwidth 962 Hz/Px). T2 maps were generated after acquiring three single-shot gradient echo images (TE 1.32 ms, flip angle = 12°; voxel size 1.9 x 1.9 x 8.0 mm, bandwidth 1184 Hz/Px with T2 preparation times of 0, 30, and 55 ms). A 5(3)3-modified Look-Locker sequence was used for T1 mapping, (TR/TE 281/1.12 ms, flip angle 35°, voxel size 1.4 x 1.4 x 8.0 mm, bandwidth 1085 Hz/Px).

### CMR Image Analysis

All analysis was blinded and conducted with cmr^42^ (Circle CVI, Calgary, Canada). To analyse clinical CMR measures at rest, T1 and T2 maps were quantified, and left-ventricular function and feature tracking strain analysis at rest was performed on the full short axis (SAX) stack cines. For the analysis of images acquired during the breathing maneuver stimulus, the relative changes of OS signal intensity (SI) in end-systolic frames, and myocardial strain as measured by CMR-FT were acquired from the same OS cines. For CMR-FT analysis, each cardiac cycle was assessed for circumferential peak strain (PS), time to PS (TTP), and peak early diastolic strain rate (dSR). Measurements for the radial orientation are provided in the supplement. All parameters were determined and reported for global measurements and for the American Heart Association segment model.

### Coronary Angiography Analysis

Independent readers analyzed the angiography images to quantify the percent diameter of the stenosis. Afterwards to allocate the coronary angiogram findings to the regional CMR analysis, the angiography readers classified each myocardial segment into one of three categories based on the individual coronary anatomy: (1) territories subtended to a current stenosis (QCA > 50%; post-stenotic) that had no previous reperfusion treatment, (2) territories revascularized at the initial angiography by coronary stenting (reperfused), or (3) territories perfused by a patent native coronary artery (remote). This allocation was primarily based on the classical perfusion territories (19), with the impact on basal, mid and apex slices determined by the proximal hierarchy of the lesions. A figure detailing this allocation protocol can be visualized in the publication by Fischer et al. (20). The angiographic readers then adjusted the territory classification based on individual coronary anatomy and dominance (17). The segmental CMR values then were averaged according to the angiography classification.

### Statistical Analysis

Continuous data are reported as mean ± standard deviation (SD), categorical data as frequency and percentage. Statistical analysis compared datasets acquired at three time points: at rest, immediately post-hyperventilation, and closest to 30 s of apnea. Myocardial oxygenation response to hyperventilation is given as percent change of OS-SI relative to the image at resting conditions. For the apnea-provoked response at 30 s, the first image acquired during full breath-hold was used as reference. Strain parameters are reported as Δ-change. First, the global myocardial response was compared between participant groups using a linear regression model accounting for sex and age as covariates. Strain data from the three time points were compared within-group only, in order to assess whether strain changed globally, or within the respective territory during the breathing maneuvers. Here, a mixed-effects model was used, accounting for repeated measures and with Tukey's *post-hoc* analysis if applicable. Thereafter, both strain (Δ) and myocardial oxygenation responses (%) were compared for each breathing maneuver step. Pearson's correlation coefficients were used to investigate the association of strain and OS-SI with mapping values.

Receiver operating characteristic curves (ROC) were used to calculate the discriminating ability of imaging parameters for the detection of post-stenotic myocardium defined by angiography. This was performed first for traditional CMR measures acquired at rest including T1 and T2 mapping, ejection fraction, and circumferential peak strain (PS_SAXStack_). ROC curves were then created for both the myocardial oxygenation response and the CMR-FT measured from the OS cine at the end of apnea. A curve combining both features at the end of the stimulus was created using binary logistic regression. Area under the curve (AUC) between correlated curves was compared using the Hanley and McNeil test.

For validation purposes, global and regional strain measurements from all the resting OS cines of the CAD patients (*n* = 25) were compared to strain measurements of the function stack cines using a two-way mixed intra-class correlation (ICC) for absolute agreement. ICC was further calculated for inter-observer agreement with a second blinded reader (*n* = 25). Statistical analyses were performed with GraphPad Prism version 9.0 (GraphPad Software, La Jolla California USA) and *R* software (version 3.5.0, R Foundation for Statistical Computing, Vienna, Austria). Results were considered statistically significant at a two-tailed value *p* < 0.05.

## Results

### Data Inclusion

One patient was excluded from analysis due to poor triggering in the CMR images. Specifically for the OS cine, FT and OS data was available from all the remaining 25 patients, and regionally 92% of the segments could be analyzed for peak strain and time to peak strain, with all exclusions due to poor plane position and no individual segments excluded for tracking issues. Diastolic strain rate could be acquired in 81% of segments, and myocardial oxygenation in 92%.

### Participant Characteristics

Baseline characteristics are provided in Table 1. All patients had at least one stenosed coronary vessel with QCA > 50%, with an average diameter stenosis of 67 ± 16%. In the CAD patients, a significant lesion was either in the proximal (68%) or mid (32%) portion of the relevant coronary artery (Table 2). As a result, when translating to the AHA segmentation for the CMR images, all patients had at least one segment in the mid-slice classified as post-stenotic territory, with a median of 5.0 [3.5–6.0] of the 16 myocardial AHA segments defined as post-stenotic when considering the entire ventricle. A PCI procedure was performed during the initial visit in 20 patients (80%).

**Table 1:** Patient characteristics.

**Demographics**	**Healthy volunteers (*n* = 12)**	**Patients (*n* = 25)**
Age (years)	56 ± 5	65 ± 9^*^
Sex (female)	4 (33%)	3 (12%)
Body mass index (kg/m^2^)	24.4 ± 2.1	28.0 ± 4.6^*^
Body weight (kg)	72.5 ± 10.4	85.3 ± 16.2^*^
**Global left ventricular CMR measures**		
Mass index (g/m^2^)	75 ± 15	68 ± 10
End-diastolic volume index (ml/m^2^)	86 ± 16	72 ± 15
End-systolic volume index (ml/m^2^)	32 ± 7	27 ± 11
Stroke volume index (ml/m^2^)	54 ± 11	44 ± 11
Ejection fraction (%)	63 ± 5	62 ± 10
Cardiac index (L/min/m^2^)	3.1 ± 0.7	2.9 ± 0.8
Peak strain_SAXstack_ (%)	−20.4 ± 3.6	−19.3 ± 1.9
Native T1 mapping (ms)	1,209 ± 41	1,228 ± 51
T2 mapping (ms)	39.2 ± 1.8	39.6 ± 2.4
**Coronary risk factors**		
Dyslipidemia	–	17 (68%)
Hypertension	–	14 (56%)
Diabetes mellitus	–	8 (32%)
Sleep apnea syndrome	–	3 (12%)
**Medication**		
Aspirin	–	25 (100%)
Statins	–	22 (88%)
Dual anti-platelet therapy	–	21 (84%)
Beta-blockers	–	18 (72%)
Angiotensin-converting enzyme inhibitors	–	9 (36%)
Angiotensin receptor blocker	–	7 (28%)
Calcium channel blocker	–	4 (16%)

*Baseline characteristics of the study subjects at the time of CMR exam. Data reported as mean ± SD or n (%)*.

* *p < 0.05 between groups*.

**Table 2:** Coronary angiography.

	**Patients (*n* = 25)**
Maximum diameter stenosis (%)	67 ± 16
>50% diameter stenosis*	
Left anterior descending artery	13 (52%)
Circumflex artery	6 (24%)
Right coronary artery	10 (40%)
**Proximal involvement** ^** 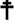 **^	
Proximal	17 (68%)
Mid	8 (32%)
Distal	0 (0%)
**Post-stenotic AHA myocardial segments**	
Whole heart (/16)	5.0 [3.5–6.0]
Basal (/6)	2.0 [0.0–2.0]
Mid (/6)	2.0 [2.0–2.0]
Apex (/4)	1.0 [1.0–2.0]
**Prior PCI**	
Left anterior descending	8 (32%)
Left circumflex	2 (8%)
Right coronary artery	10 (40%)
None	5 (20%)
**Reperfused AHA myocardial segments**	
Whole heart (/16)	5.0 [2.5–6.0]
Basal (/6)	2.0 [0.0–2.0]
Mid (/6)	2.0 [0.5–2.0]
Apex (/4)	1.0 [0.5–2.0]

*Coronary status at the time of the cardiovascular magnetic resonance exam. Data is reported as mean ± SD, n (%) or as median [interquartile range]. The number of post-stenotic and reperfused myocardial AHA segments correspond to the classification of myocardial territories derived from the coronary angiography. AHA, American Heart Association; PCI, percutaneous coronary intervention. *A significant stenosis in multiple vessels per patient was possible. ^
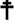
^Location of the most proximal significant lesion (>50% diameter stenosis)*.

Standard left ventricular function measures of ejection fraction (63 ± 5 vs. 62 ± 10%, *p* = 0.965) and cardiac index (3.1 ± 0.7 vs. 2.9 ± 0.8 L/min/m^2^, *p* = 0.745) acquired at resting conditions did not differ between controls and patients. Nor did global peak circumferential strain acquired from the standard short axis stack differ between the groups (PS_SAXStack_−19.3 ± 1.9 vs.−20.4 ± 3.6 %, *p* = 0.279). Similarly, there was no difference in the tissue characterization in global native T1 (1209 ± 41 vs. 1228 ± 51 ms, *p* = 0.055) and T2 mapping (39.2 ± 1.8 vs. 39.6 ± 2.4 ms, *p* = 0.642) between groups. Nor were there regional differences between post-stenotic, reperfused and remote territory for either native T1 (1229 ± 53, 1222 ± 66, and 1223 ± 52 ms, *p* = 0.857) or T2 (40.4 ± 3.5, 39.3 ± 1.9, and 39.4 ± 2.9 ms, respectively, *p* = 0.654).

### Validation of Strain Analysis From ECG-Triggered OS Cines

For global and post-stenotic myocardium strain results from OS cines there was moderate to good intra-class correlation with results derived from the function stack for both global and territorial measurements (*n* = 25, Supplementary Table 1). Global PS showed best agreement (ICC: 0.900, *p* < 0.001) between sequence types. The ICC for inter-observer agreement for PS from the OS cines was excellent at 0.907 (*p* < 0.001, *n* = 25).

### Response to Breathing Maneuvers in Healthy Subjects

**Figure 2:**
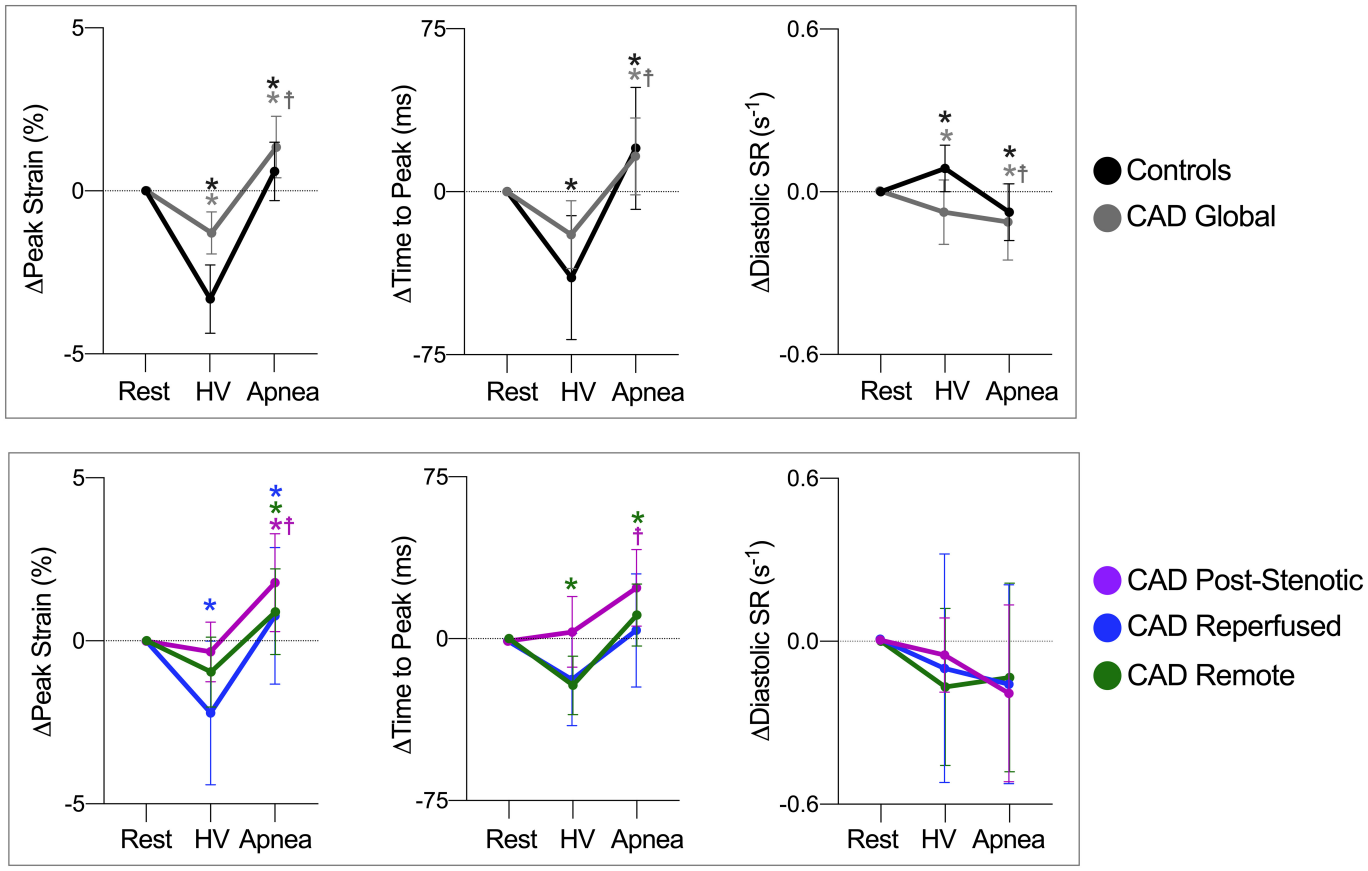
Response of circumferential strain parameters to breathing maneuvers. Data are mean (±95%CI) changes of global peak strain, time to peak strain and early diastolic strain rate (dSR) after hyperventilation (HV) and apnea. Global myocardial changes are shown on the top row for healthy controls (black) and coronary artery disease CAD patients (gray), and for angiography defined myocardial territories on the bottom. *p < 0.05 from the previous step, ^
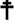
^*p* < 0.05 at rest vs. apnea.

**Figure 3:**
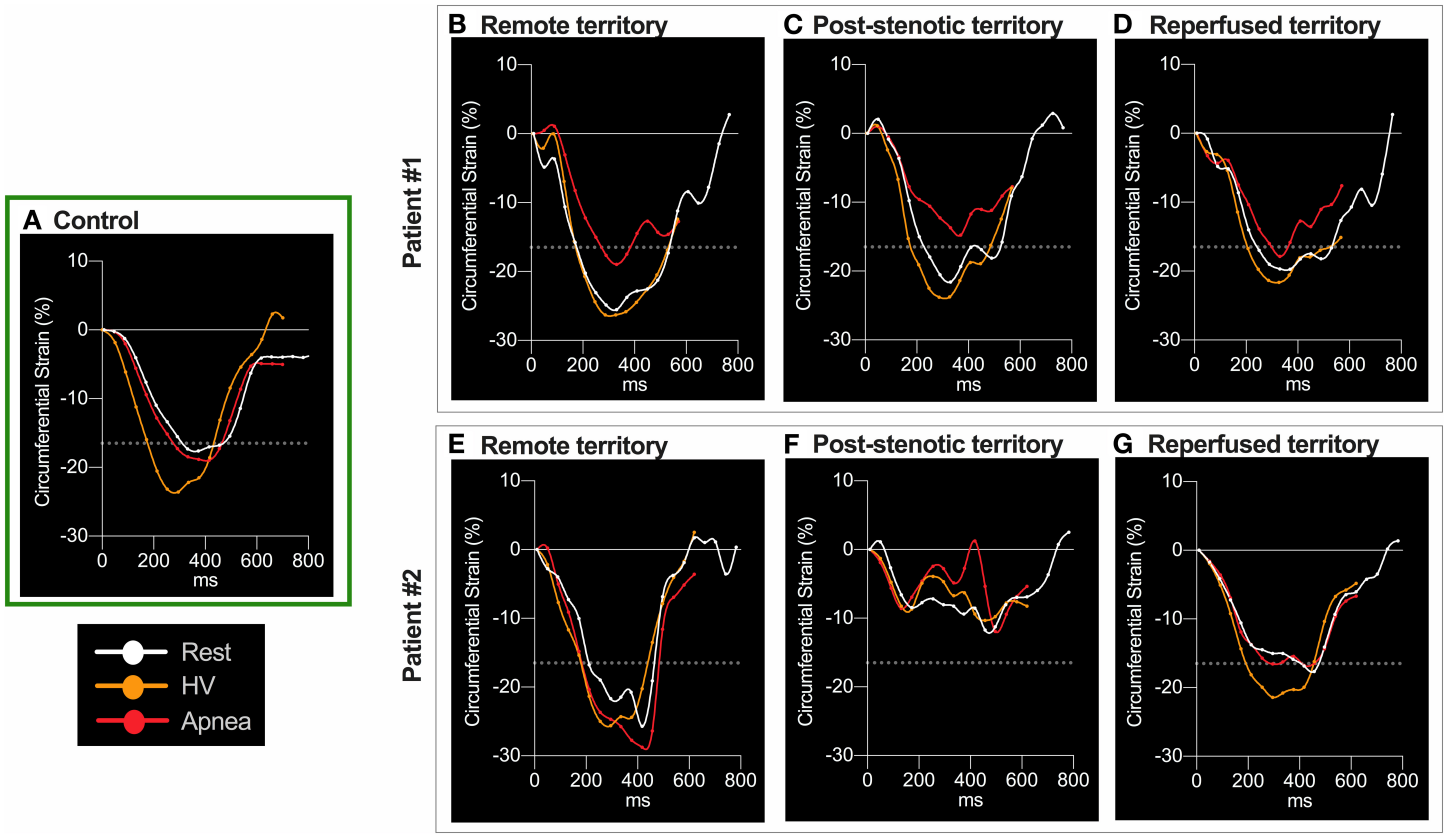
Circumferential strain curve at the different breathing maneuver steps. Circumferential strain during the cardiac cycle for three subjects. In the healthy control participant **(A)**, hyperventilation (HV, orange) improved peak strain (PS), seen by the more negative values, and shortened time to peak strain (TTP) in comparison to resting conditions (white). Both normalized with apnea (red). The dotted line denotes the level of 1 SD below mean PS of control subjects at rest (-16.5%). Values lower than−16.5% were regarded as normal peak circumferential shortening in strain. Patient 1: All three territories had normal PS at rest. HV improved PS without altering TTP. In remote myocardium **(B)** PS was not attenuated beyond−16.5% and TTP remained unchanged during apnea. In post-stenotic territory regional T1 was 1153ms and T2 was elevated at 45.2ms. Here apnea aggravated PS more severely to a subnormal level **(C)** and prolonged TTP. The increase in TTP was representative for post-stenotic myocardium in CAD. Reperfused territory experienced stable TTP and less apnea-induced attenuation of PS, still being in the normal range **(D)**. In the reperfused segments normal native T1 (1120ms) and slightly increased T2 (42.2ms) were found in this patient 33 days after NSTEMI. Patient 2: PS in remote territory remained relatively unaffected by the breathing maneuver **(E)**. In post-stenotic **(F)** territories PS was abnormal already at rest and also during provocation. In contrast to the majority of patients, here apnea even provoked severe systolic function in the post-stenotic segments (T1, 1273 ms; T2, 35.9 ms). In territories reperfused 36 days after an NSTEMI **(G)**, PS was borderline with no attenuation due to the breathing maneuvers (T1, 1252 ms; T2, 36.5 ms).

Myocardial oxygenation response in healthy subjects was characterized by a reduction of myocardial SI after 60 s of hyperventilation (-10.2 ± 8.3%) and an increase (+9.1 ± 5.3%) after 30 s of apnea. At the end of hyperventilation, PS was significantly augmented compared to baseline (Figure 2). This was accompanied by a shortening of TTP and acceleration of dSR. These changes returned to baseline during apnea (Figure 3A, Supplementary Figures 1A, 2). The findings for when TTP was additionally corrected for heart-rate are shown in Supplementary Figure 3, Figure 3A shows a homogenous oxygenation and strain response of a healthy subject.

### Global Response in CAD Patients

In response to hyperventilation, CAD patients showed a global myocardial OS-SI response of −5.8 ± 9.6 % (*p* = 0.085 vs. controls), and in response to apnea a smaller global OS-SI increase (+2.7 ± 4.8 %, *p* < 0.001 vs. controls), when compared to healthy controls. Moreover, this attenuated global response was correlated with increasing T1 (r = −0.45, *p* = 0.002, Supplementary Table 3). Strain analysis of CAD patients demonstrated an attenuated hyperventilation-induced change of global PS (Figure 2) in comparison to healthy controls (Δ-1.3 ± 1.6 vs.−3.3 ± 1.6 %, *p* = 0.008). By 30 s of subsequent apnea, PS had significantly worsened beyond values at rest (*p* < 0.001). This peak strain response during apnea was also significantly attenuated in comparison to controls (Figure 2, *p* = 0.025). Global TTP of patients did not respond significantly to hyperventilation, in contrast to TTP of healthy controls. Apnea however prolonged global TTP in patients (*p* = 0.002). Global early dSR was attenuated both during hyperventilation and during apnea in CAD patients (*p* = 0.014 and *p* = 0.012, respectively). Radial strain data are given in Supplementary Figure 2, Supplementary Table 2. With the breathing maneuver stimulus patients reported the onset of minor adverse effects such as dizziness, dry mouth and tingling in the digits that dissipated upon termination of the maneuver. No patients reported any angina or significant discomfort.

### Regional Myocardial Oxygenation and Function in CAD Patients

**Figure 4:**
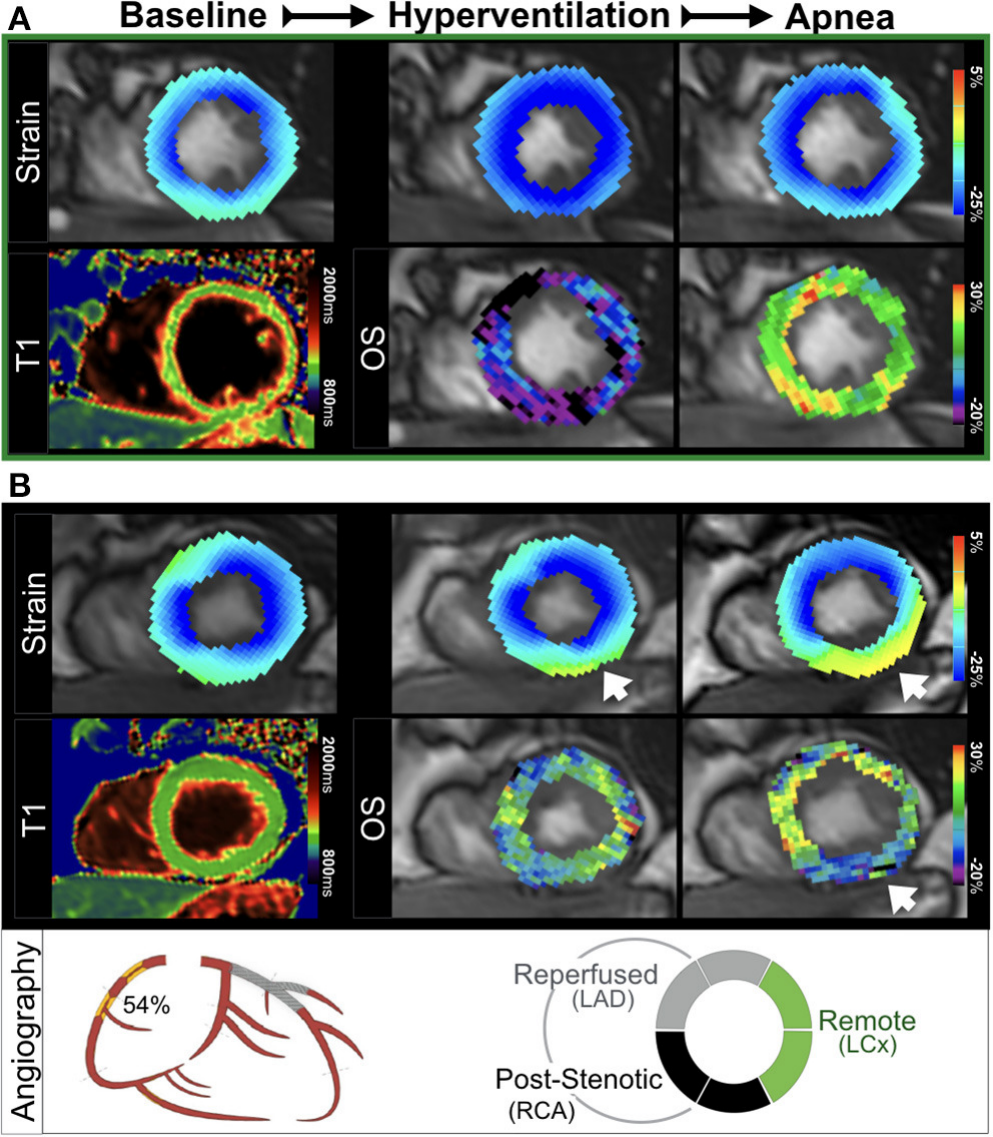
Ventricular dysfunction and myocardial deoxygenation induced by apnea. **(A)** In a healthy control, hyperventilation (HV) augmented peak strain (PS) homogenously to more negative values. This was accompanied by a HV-induced decrease in OS-SI (blue), followed by an increase of oxygenation (green-yellow) with apnea and normalization of (PS), which is the physiologic response to the breathing maneuver sequence. **(B)** In a patient (Figure 2, patient 1) with an RCA stenosis (QCA 54%) and a stented LAD, a normal PS was observed in the myocardium at rest. Hyperventilation induced a mild peak strain abnormality in the inferior wall that worsened in apnea (yellow, arrow). This functional reduction was colocalized with apnea-induced regional deoxygenation in post-stenotic myocardium (blue-purple). There was no increased native T1 found in this patient.

**Figure 5:**
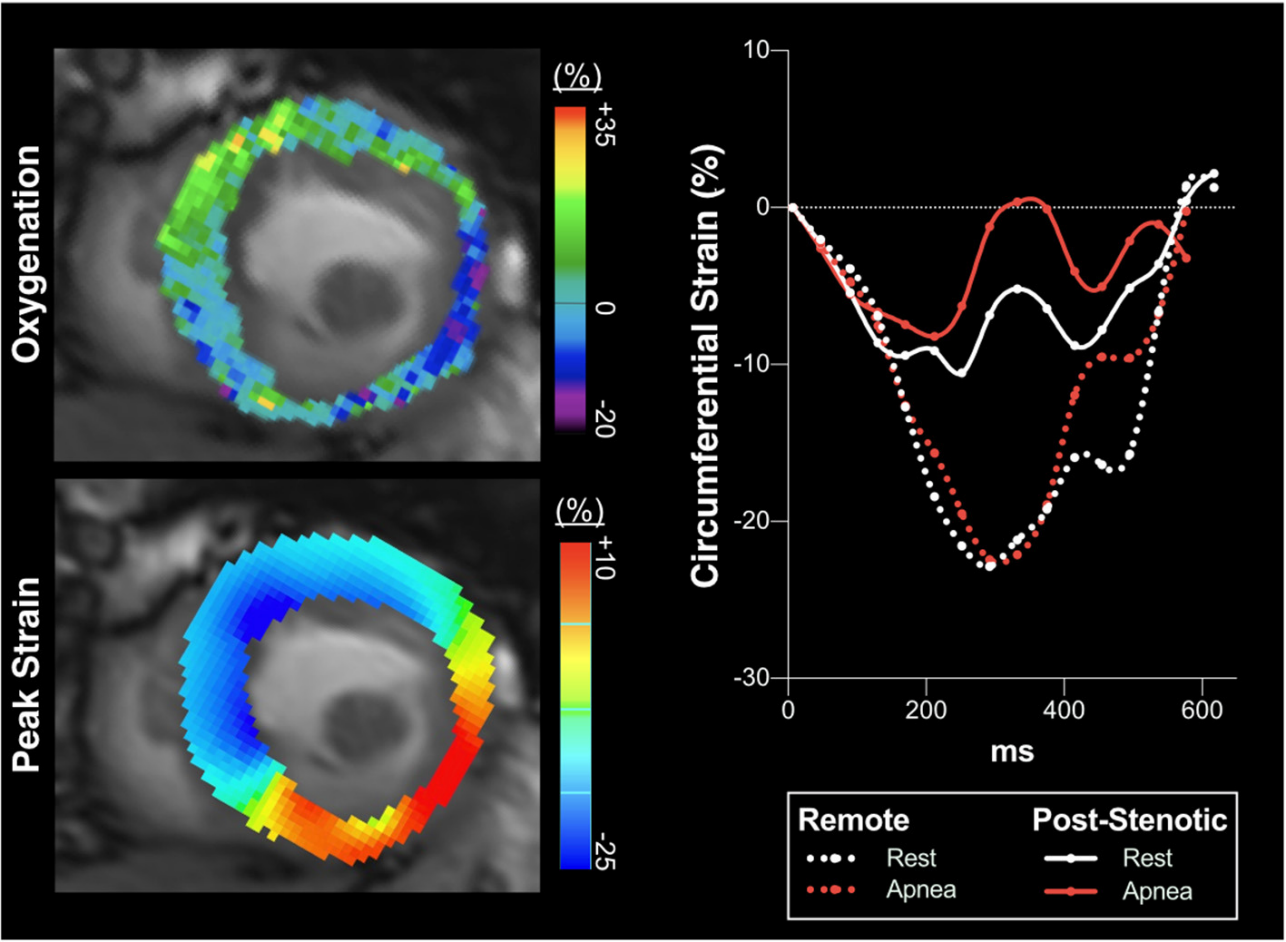
Circumferential strain over the cardiac cycle. In a patient with a full occlusion of the right coronary artery partially compensated for by collaterals, deoxygenation occurs in the inferoseptal, inferior and lateral myocardial segments in response to brief intentional apnea seen with OS-CMR (upper left panel, mid-ventricular short axis slice, blue indicating oxygenation deficits, while green shows a normal response). This is accompanied by impaired peak strain (PS, yellow-orange) of the very same segments during apnea (lower left panel, circumferential strain CMR-FT analysis), while remote healthy myocardial segments display a normal physiologic response. The right panel shows strain over the cardiac cycle averaged for the post-stenotic and remote territories defined by the angiography analysis. While remote myocardium at apnea slightly increased (PS) beyond the resting baseline, the angiographically defined post-stenotic myocardium in the inferoseptal and inferior wall showed an averaged pronounced attenuation of PS represented by less negative strain values indicating aggravating severe hypokinesia to regional dyskinesia (seen also in the left lower panel) of these segments triggered by myocardial deoxygenation during apnea. This patient did not have any reperfused territory. Both native T1 (1381 ms) and T2 (44.2 ms) mapping were elevated in the post-stenotic territory.

Hyperventilation did not induce oxygenation differences between post-stenotic (-5.5 ± 10.5 %), remote myocardium (-7.1 ± 11.3%) and reperfused territories (-3.3 ± 8.4%, *p* = 0.350). The subsequent apnea consistently provoked regional oxygenation heterogeneities in post-stenotic (+1.6 ± 3.9%, *p* = 0.004) and reperfused territories 1.3 ± 6.0%; *p* = 0.024) compared to the response in remote territories (+4.9 ± 5.7%). Strain and myocardial oxygenation responses of territories and provocation maneuvers steps are detailed in Supplementary Table 2 and depicted in Figures 2–5. All territories exhibited attenuated PS during apnea when compared to post-hyperventilation (Figure 1, *p* < 0.05), while post-stenotic myocardium was the only territory to have a further reduction in strain with apnea beyond baseline (*p* = 0.004). Similarly, post-stenotic myocardium was the only territory to have a significantly prolonged TTP (rest: 319 ± 46, hyperventilation: 324 ± 59, apnea: 343 ± 58, *p* = 0.022 vs. rest). This can also be seen in Supplementary Table 2. Remote myocardium showed significantly shortened TTP after hyperventilation (*p* = 0.008) with recovery to baseline during apnea (*p* < 0.001), which was comparable to the respective global response of healthy controls, while no response in TTP was observed in reperfused myocardium. There were no significant territorial changes or differences in dSR. Medications taken by the patients such as beta-blockers did not have a statistical impact on the post-stenotic myocardial oxygenation response or on the strain parameters measured during hyperventilation or apnea (Supplementary Figure 4).

### Resting vs. Post-stimulatory Discrimination of Post-stenotic Territory

**Figure 6:**
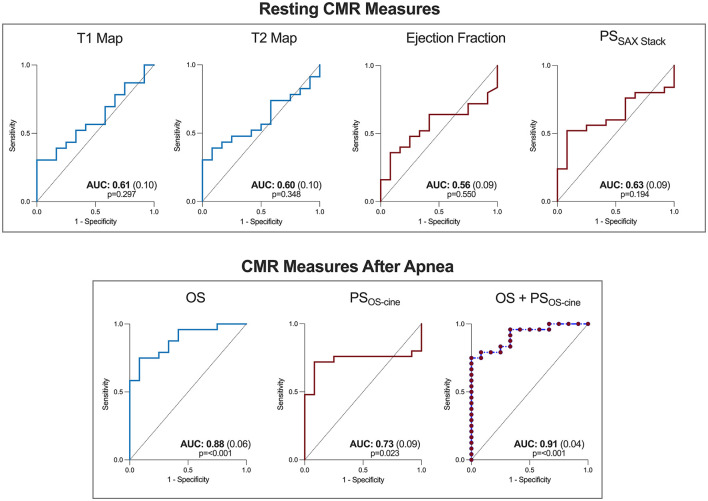
Combined oxygenation-sensitive and feature tracking analysis improves determination of post-stenotic myocardium. Receiver operating characteristic curves are shown for the CMR parameters at resting conditions (top) for both tissue characterization sequences (blue) and functional measurements (red). The bottom row depicts the oxygenation-sensitive (OS) response after 30s of apnea, and the feature tracking peak strain (OS) measurements acquired from the same image, along with a combined predicted curve combining both tissue and functional measures. AUC, Area under the curve (standard error).

The ROC analysis in Figure 6 shows, that both tissue characterization and functional CMR measurements acquired in normal resting conditions were not able to discriminate post-stenotic territory (*p* > 0.05). For CMR measures obtained with the breathing maneuver stimulus, both OS-CMR (AUC: 0.88, SE: 0.06, *p* < 0.001) and PS measured from the OS-cine (AUC: 0.73, SE: 0.09, *p* = 0.023) were able to individually detect post-stenotic territories at the end of apnea. The combination of the two measures of OS-CMR and PS yielded an AUC of 0.91 (SE: 0.04, *p* < 0.001) and was significantly better at defining post-stenotic territories than the post-apneic PS measurement alone (*p* = 0.036).

## Discussion

The major finding of this study is that in patients with chronic coronary syndromes and primarily intermediate coronary stenoses, regional impairment of myocardial kinetics provoked by voluntary breathing maneuvers is detected by feature tracking strain analysis on oxygenation-sensitive (OS) cine acquisitions. Moreover, myocardial oxygenation is compromised in these same territories, with the combined analysis of tissue and wall function improving the detection of post-stenotic myocardium. In fact, this could only be differentiated with the breathing maneuver as traditional contrast-free tissue and functional measures at rest were unable to discriminate post-stenotic myocardium of primarily intermediate-grade stenoses from healthy controls with similar age. These measurements of myocardial oxygenation and strain can be simultaneously obtained from the same set of OS cines, ensuring both measurements are of the same territory and represent the same timepoint during the breathing maneuver provocation stimulus.

### Oxygenation and Strain Response to Hyperventilation

As previously reported, healthy participants respond to hyperventilation with decreasing myocardial oxygenation, and to apnea with increased tissue oxygenation (16, 17, 21). Although not completely understood, multiple mechanisms can simultaneously occur during breathing maneuvers that lead to this physiologic response with a key pathway likely driven by hypocapnic vasoconstriction and hypercapnic vasodilation of coronary vasculature (13, 16). The increase in heart rate which accompanies hypocapnic vasoconstriction can further increase oxygen consumption and promote deoxygenation (12, 22, 23). Enhanced contractility and heart rate acceleration from sympathetic stimulation can lead to the increase of PS and shortening of TTP observed in healthy subjects (24). For this combined breathing maneuver technique, hyperventilation primarily serves as the preparatory phase to induce a state of hypocapnia, which will result in a greater range of CO_2_ manipulation to be observed with the apnea and also allow the patient to maintain a longer breath-hold. The second phase of the combined breathing maneuver, the apnea component, is the focus of the analysis where oxygenation and functional deficits are more consistently revealed.

### Oxygenation and Strain Response to Apnea

During apnea arterial carbon dioxide partial pressure rises and heart rate slows, promoting vasodilation and enhancing myocardial oxygenation in healthy myocardium (12, 16). As even breath-holds as short as 10–15 s have been reported to lead to a detectible myocardial and cerebral hyperemic response (17, 25). A combined function of the respiratory, cardiac parasympathetic and vasomotor centers likely play a supporting role in improving myocardial perfusion (26). In healthy participants post-hyperventilation apnea was accompanied by luxury myocardial oxygenation and return of all strain parameters to resting values. However, in our patient group, a detailed regional analysis showed that these patients exhibited heterogenous territorial responses of both myocardial oxygenation and strain indicating that respiratory provocation maneuvers act differently on remote, reperfused and post-stenotic myocardium. Specifically, myocardial deoxygenation developed in the post-stenotic segments during apnea. Coronary arteries affected by a fixed epicardial stenosis exhibit a post-stenotic compensatory vasodilation, exploiting the vasodilatory range. Post-stenotic blood flow becomes pressure dependent and vasodilating stimuli such adenosine or apnea cannot increase blood flow further, which results in an attenuated or blunted increase in SI in OS-CMR sequences (5). In the presence of compensatory post-stenotic dilation and high reported post-stenotic pCO_2_ levels in CAD patients, the post-stenotic vasculature cannot dilate further and the observed regional heterogeneity may be further explained by redistribution of blood flow and possibly inter-coronary steal away from territory distal to a fixed stenosis, which may lead to a net decrease in OS-SI (12, 17, 27).

Importantly, myocardial deoxygenation does not reflect ischemia *per se*. As long as oxygen supply exceeds myocardial demand, the observed deoxygenation only delineates myocardium at risk for ischemia (28). If the oxygen demand is not matched by the supply, ischemia and myocardial dysfunction are the consequence. The worsened myocardial kinetics in post-stenotic myocardium in the CAD group occurred in the same myocardial tissue that desaturated during the breathing maneuver, hinting to early ischemic sequelae. Post-stenotic myocardium showed an increase of mean TTP along with attenuated peak strain from baseline to apnea, which may be explained by an increasingly compromised myocardial oxygenation. This was not seen in remote or reperfused territories. During isovolumetric contraction, pressure in the LV cavity increases when segments with sufficient oxygen supply contract in synchronized fashion (24, 29). Segments with compromised myocardial oxygenation are prone to delayed contraction after opening of the aortic valve compared to unaffected segments. This is known as post-systolic shortening, indicated by heterogenous TTP between segments, which has a high sensitivity to ischemia (30, 31).

### Diastolic Function

Strain assessments can also provide details on diastolic dysfunction, which is an early harbinger of ischemia, preceding systolic dysfunction, electrocardiogram abnormalities and other ischemic sequelae (32). Early peak diastolic strain rate (dSR) at CMR-FT reports the deformation rate during the active, energy dependent part of ventricular relaxation (33). Accordingly, step-wise global deceleration of early diastolic strain across the breathing maneuvers was observed, and this was associated with increased T2 burden. Edema is also linked with ventricular stiffness and when myocardial edema aggravates, the rise of interstitial pressure reduces ventricular chamber compliance (34, 35). While T2 is not often chronically elevated, edema at the time of CO_2_ challenges may predispose cardiac patients to diastolic dysfunction. However, the use of regional diastolic strain rate by FT is limited and still developing, as observed by the variation observed in Figure 2.

### Imaging the Different Features of the Ischemic Sequalae

The onset of acute cardiac ischemia is a well described progression of abnormal cardiovascular features (2). The order of these features has often been described as a constellation or cascade, in which after the onset of a negative imbalance in myocardial oxygen supply and demand, myocardial perfusion abnormalities are observed early in the cascade, followed by ventricular diastolic and systolic dysfunction, before culminating in electrocardiogram abnormalities and symptoms of myocardial ischemia (3, 36). In an animal study, the spatiotemporal difference observed between perfusion deficits and ventricular dysfunction varied between the subjects based on the degree of disease (37). This variation highlights the importance of using imaging to target multiple stages of this cascade to detect the onset of ischemia in the early stages. In our findings, the presence of diastolic and systolic dysfunction as early sequelae of flow maldistributions in relation to our measured decreases in myocardial oxygenation is suggestive that our breathing maneuver may have triggered inducible ischemia. The fact that short breathing maneuver challenges can trigger this ischemic sequelae may be of concern to situations where the heart is under stress. This may occur if these patients undergo exertion, or during other medical procedures that expose a patient to multiple stimuli. An example may be during general anesthesia, which is an environment where fluctuations in blood gases and respiratory rates frequently occur in combination with other stimuli. Consequently, these CCS patients could be at risk of peri-operative induced ischemia, despite normal resting function.

### Diagnostic Potential

While in echocardiography myocardial strain analysis is already firmly established, studies reporting diagnostic and prognostic potential of resting strain measurements from CMR imaging are emerging (38–41). Strain analysis from either modality is useful to identify subtle deficiencies in regional contractile function prior to decreases in left ventricular stroke volume. Reduced myocardial strain emerged as a superior predictor of adverse outcome from myocardial infarction when compared to cardiac output data (39). Strain can be an early marker as impaired contractility in small regions may not necessarily result in globally reduced ejection fraction as long as a sufficient mass of unaffected myocardium can compensate. However, in this cohort with CCS and often intermediate angiographically defined lesions, there was no difference between groups in CMR-FT acquired from the typical short-axis stack at resting conditions, nor could it discriminate post-stenotic territory. In line with strain, other contrast-free CMR measures acquired at rest including ejection fraction and the tissue characterization sequences of T1 and T2 also did not differentiate post-stenotic territory in this small sample.

In particular for investigating ischemia, stress exams are implemented to provoke inducible ischemia. This has been performed frequently with echocardiography based strain measurements, commonly using pharmacological stimuli such as dobutamine or dipyridamole (42). CMR-FT has already demonstrated its usefulness in the detection of CAD during dobutamine stress (43). More recently, Romano et al. (44) used blunted responses of feature tracking longitudinal strain to a vasodilator stress as an independent predictor of major adverse cardiac events in patients with CAD incremental to traditional clinical risk factors or imaging results such as ejection fraction and late gadolinium enhancement. CMR is beneficial as it also investigates myocardial tissue features and vascular function. First pass perfusion scans (5) and native T1 stress mapping both show perfusion deficits under vasodilator stress (45). However, there is a significant proportion of patients with contraindications against contrast agents and vasodilating agents. Similar to native T1 mapping and strain imaging, OS-CMR does not rely on contrast agents. Previous studies have used OS-CMR together with adenosine as a vasodilator stress (5, 46), but non-pharmacological approaches such as inhalation of CO_2_ and breathing maneuvers have been proposed as alternative vasodilator stimuli (14–16).

Provocation testing of strain parameters with simple breathing maneuvers may be another perspective for early non-invasive diagnostics, even by employing echocardiography as the more widely available imaging modality. As shown by Ochs et al. (47) by implementing strain encoded MR imaging after the same combined breathing maneuver, this group was able to detect coronary stenosis with an even higher diagnostic accuracy than adenosine-based perfusion imaging. This supports our findings about the functional impact of hyperventilation and apnea in a coronary artery disease population. Each approach has its advantages. Although strain encoded imaging is an acquisition-based technique requiring special images to be acquired during the exam, it can have a higher reproducibility than feature tracking based measurements (48). Recent publications have demonstrated that CMR-FT has a high reproducibility between readers in a patient cohort, especially for circumferential parameters in comparison to longitudinal markers (49). As mentioned above, the key advantage of our technique is the ability to use CMR-FT post-processing software to simultaneously acquire myocardial oxygenation and deformation information. In Figure 6, it can be observed that the combination of oxygenation-sensitive imaging on top of CMR-FT, increased the area under the curve for detecting post-stenotic territory over CMR-FT alone. However, to image this dynamic change in myocardial function rapid acquisition was required and consequently only two short-axis views were acquired, and long-axis views were not available. In the future it would be wise to apply this technique to investigate longitudinal strain as well, as ischemia is likely to first arise in the subendocardium. Since this myocardial layer is composed primarily of longitudinal fibers, longitudinal strain may have the potential to detect inducible ischemia even earlier. Future work needs to confirm the diagnostic utility of these techniques for non-pharmacologic and endogenous stress testing. Further work can investigate the comparison between strain response of orientations. Importantly, the circumferential strain analysis allows a better matching to the tissue characterization sequences often acquired in a short-axis view.

### Limitations

As this was a contrast-free exam, our study is limited by the lack of late gadolinium enhancement (LGE) and extracellular volume mapping (ECV). Thus, the presence or absence of scar cannot be confirmed in this cohort, nor can the impact of scar on the strain response to breathing maneuvers be determined at this point. In patients with myocardial infarction, resting strain has been reported to be related to the extent of LGE (39). However, in a heart failure cohort without infarct patients, there was no association of resting strain with LGE, rather a correlation to T2 mapping and the OS response with apnea (50). This will have to be investigated in the future to determine if infarcted territory impacts both resting strain, and the dynamic response to a stimulus. Of note, there was no significant difference in native T1 or T2 between the allocated territories in our cohort. The role of native T1 and CMR-FT may help in the development of contrast free protocols (45, 47, 51). Our model did not take the hemodynamic significance of a stenosis and its possible collateralization into account. Our enrolment procedures were based on anatomical measures, as it was a marker available for all patients and is a measurement that is not limited by complex and serial lesions. FFR measurements to address this issue were not available for this study, and it would be important in the future to investigate the heterogeneity in strain responses in relation to the hemodynamic significance of the stenosis. This study had a small sample size, and utility of this technique needs to be validated in larger cohorts, in single and multi-vessel disease, and with a greater range of degrees of stenoses.

The fact that majority of the enrolled CAD patients were under chronic medication with beta-blockers and ACE inhibitors could confound the heart rate increase, oxygenation and strain response observed in the patient group. In this population, patient medications were not statistically associated with the oxygenation or strain results during hyperventilation or apnea. Pharmacological beta-blockade would not explain or confound the inter-territorial differences seen within our CAD patient cohort. Similar to our findings, it has been demonstrated that beta-blocker therapy did not impact myocardial perfusion imaging with adenosine (52), or the heart rate response to hyperventilation (23). Nevertheless, little is known about the effect of beta-blockers and breathing maneuvers on myocardial strain and oxygenation in larger samples.

## Conclusion

In myocardium subtended to an anatomically defined intermediate-grade coronary stenosis of patients with chronic coronary syndromes, an oxygenation-sensitive (OS)-CMR cine acquisition during a breathing maneuver can simultaneously unmask an impaired vascular and functional response. The detection of post-stenotic myocardium was improved with a combined approach of measuring an attenuated myocardial oxygenation reserve along with CMR feature-tracking. Furthermore, peak strain is attenuated and time to peak strain is prolonged exclusively in post-stenotic segments at apnea. These findings may be indicative of inducible early myocardial ischemia.

## Data Availability Statement

The raw data supporting the conclusions of this article will be made available by the authors, without undue reservation.

## Ethics Statement

The study protocol was approved by the Ethics Board of the Canton of Bern. The patients/participants provided their written informed consent to participate in this study.

## Author Contributions

DG, KF, BE, and BJ: conceptualization and methodology. DG, KF, BS, KY, YU, ZZ, CB, TO, MB, CG, and LR: investigation and analysis. BS, KF, and DG: original manuscript draft. KY, YU, BJ, TO, MB, CG, HvT-K, LR, BE, and DG: manuscript revision and editing. KF: visualization. DG, BE, HvT-K, and LR: supervision. DG and BE: project administration. All authors contributed to the article and approved the submitted version.

## Funding

This work was supported by institutional research funds of the Department of Anaesthesiology and Pain Medicine at Inselspital, Bern University Hospital, Switzerland, and by the European Society of Anaesthesiology Research Project Grant.

## Conflict of Interest

The authors declare that the research was conducted in the absence of any commercial or financial relationships that could be construed as a potential conflict of interest.

## Publisher's Note

All claims expressed in this article are solely those of the authors and do not necessarily represent those of their affiliated organizations, or those of the publisher, the editors and the reviewers. Any product that may be evaluated in this article, or claim that may be made by its manufacturer, is not guaranteed or endorsed by the publisher.

## References

[1] Knuuti J Wijns W Saraste A Capodanno D Barbato E Funck-Brentano C. 2019 ESC Guidelines for the diagnosis and management of chronic coronary syndromes: the task force for the diagnosis and management of chronic coronary syndromes of the European society of cardiology (ESC). Eur Heart J. (2020) 41:407–77. 10.1093/eurheartj/ehz425 31504439

[2] Nesto RW Kowalchuk GJ. The ischemic cascade: temporal sequence of hemodynamic, electrocardiographic and symptomatic expressions of ischemia. Am J Cardiol. (1987) 59:C23–30. 10.1016/0002-9149(87)90192-5 2950748

[3] Maznyczka A Sen S Cook C Francis DP. The ischaemic constellation: an alternative to the ischaemic cascade-implications for the validation of new ischaemic tests. Open Heart. (2015) 2:e000178. 10.1136/openhrt-2014-000178 26196015 PMC4505364

[4] Stillman AE Oudkerk M Bluemke DA de Boer MJ Bremerich J Garcia EV. Imaging the myocardial ischemic cascade. Int J Cardiovasc Imaging. (2018) 34:1249–63. 10.1007/s10554-018-1330-4 29556943

[5] Luu JM Friedrich MG Harker J Dwyer N Guensch D Mikami Y. Relationship of vasodilator-induced changes in myocardial oxygenation with the severity of coronary artery stenosis: a study using oxygenation-sensitive cardiovascular magnetic resonance. Eur Heart J Cardiovasc Imaging. (2014) 15:1358–67. 10.1093/ehjci/jeu138 25104812

[6] Pauling L Coryell CD. The magnetic properties and structure of hemoglobin, oxyhemoglobin and carbonmonoxyhemoglobin. Proc Natl Acad Sci U S A. (1936) 22:210–6. 10.1073/pnas.22.4.210 16577697 PMC1076743

[7] Ogawa S Lee TM Kay AR Tank DW. Brain magnetic resonance imaging with contrast dependent on blood oxygenation. Proc Natl Acad Sci USA. (1990) 87:9868–72. 10.1073/pnas.87.24.9868 2124706 PMC55275

[8] Kim S-G Ogawa S. Biophysical and physiological origins of blood oxygenation level-dependent fMRI signals. J Cereb Blood Flow Metab. (2012) 32:1188–206. 10.1038/jcbfm.2012.23 22395207 PMC3390806

[9] Wacker CM Bock M Hartlep AW Beck G van Kaick G Ertl G. Changes in myocardial oxygenation and perfusion under pharmacological stress with dipyridamole: assessment using T^*^2 and T1 measurements. Magn Reson Med. (1999) 41:686–95. 10.1002/(sici)1522-2594(199904)41:4<686::aid-mrm6>3.0.co;2-9 10332843

[10] Guensch DP Michel MC Huettenmoser SP Jung B Gulac P Segiser A. The blood oxygen level dependent (BOLD) effect of in-vitro myoglobin and hemoglobin. Sci Rep. (2021) 11:11464. 10.1038/s41598-021-90908-x 34075096 PMC8169704

[11] Fischer K Neuenschwander MD Jung C Hurni S Winkler BM Huettenmoser SP. Assessment of myocardial function during blood pressure manipulations using feature tracking cardiovascular magnetic resonance. Front Cardiovas Med. (2021) 8:1353. 10.3389/fcvm.2021.743849 34712713 PMC8545897

[12] Fischer K Guensch DP Shie N Lebel J Friedrich MG. Breathing maneuvers as a vasoactive stimulus for detecting inducible myocardial ischemia - an experimental cardiovascular magnetic resonance study. PLoS ONE. (2016) 11:e0164524. 10.1371/journal.pone.0164524 27741282 PMC5065132

[13] Crystal GJ. Carbon dioxide and the heart: physiology and clinical implications. Anesth Analg. (2015) 121:610–23. 10.1213/ANE.0000000000000820 26287294

[14] Beaudin AE Brugniaux JV Vöhringer M Flewitt J Green JD Friedrich MG. Cerebral and myocardial blood flow responses to hypercapnia and hypoxia in humans. Am J Physiol Heart Circ Physiol. (2011) 301:H1678–1686. 10.1152/ajpheart.00281.2011 21724871

[15] Yang H-J Yumul R Tang R Cokic I Klein M Kali A. Assessment of myocardial reactivity to controlled hypercapnia with free-breathing T2-prepared cardiac blood oxygen level-dependent MR imaging. Radiology. (2014) 272:397–406. 10.1148/radiol.14132549 24749715 PMC4263621

[16] Guensch DP Fischer K Flewitt JA Yu J Lukic R Friedrich JA. Breathing manoeuvre-dependent changes in myocardial oxygenation in healthy humans. Eur Heart J Cardiovasc Imaging. (2014) 15:409–14. 10.1093/ehjci/jet171 24078154

[17] Fischer K Yamaji K Luescher S Ueki Y Jung B von Tengg-Kobligk H. Feasibility of cardiovascular magnetic resonance to detect oxygenation deficits in patients with multi-vessel coronary artery disease triggered by breathing maneuvers. J Cardiovasc Magn Reson. (2018) 20:31. 10.1186/s12968-018-0446-y 29730991 PMC5937049

[18] Guensch DP Fischer K Yamaji K Luescher S Ueki Y Jung B. Effect of hyperoxia on myocardial oxygenation and function in patients with stable multivessel coronary artery disease. J Am Heart Assoc. (2020) 9:e014739. 10.1161/JAHA.119.014739 32089047 PMC7335579

[19] Donato P Coelho P Santos C Bernardes A Caseiro-Alves F. Correspondence between left ventricular 17 myocardial segments and coronary anatomy obtained by multi-detector computed tomography: an ex vivo contribution. Surg Radiol Anat. (2012) 34:805–10. 10.1007/s00276-012-0976-1 22569833

[20] Fischer K Ranjan R Friess J-O Erdoes G Mikasi J Baumann R. Study design for a randomized crossover study investigating myocardial strain analysis in patients with coronary artery disease at hyperoxia and normoxemia prior to coronary artery bypass graft surgery (StrECHO-O2). Contemp Clin Trials. (2021) 110:106567. 10.1016/j.cct.2021.106567 34517140

[21] Fischer K Guensch DP Friedrich MG. Response of myocardial oxygenation to breathing manoeuvres and adenosine infusion. Eur Heart J Cardiovasc Imaging. (2015) 16:395–401. 10.1093/ehjci/jeu202 25336541

[22] Burton DA Stokes K Hall GM. Physiological effects of exercise. Cont Educ Anaesth Crit Care Pain. (2004) 4:185–8. 10.1093/bjaceaccp/mkh050

[23] Hawkins SM Guensch DP Friedrich MG Vinco G Nadeshalingham G White M. Hyperventilation-induced heart rate response as a potential marker for cardiovascular disease. Sci Rep. (2019) 9:1–10. 10.1038/s41598-019-54375-9 31784617 PMC6884614

[24] Alexopoulos D Christodoulou J Toulgaridis T Sitafidis G Klinaki A Vagenakis AG. Hemodynamic response to hyperventilation test in healthy volunteers. Clin Cardiol. (1995) 18:636–41. 10.1002/clc.4960181109 8590532

[25] Liu H-L Huang J-C Wu C-T Hsu Y-Y. Detectability of blood oxygenation level-dependent signal changes during short breath hold duration. Magn Reson Imaging. (2002) 20:643–8. 10.1016/S0730-725X(02)00595-7 12477561

[26] Foster GE Sheel AW. The human diving response, its function, and its control. Scand J Med Sci Sports. (2005) 15:3–12. 10.1111/j.1600-0838.2005.00440.x 15679566

[27] Becker LC. Conditions for vasodilator-induced coronary steal in experimental myocardial ischemia. Circulation. (1978) 57:1103–10. 10.1161/01.CIR.57.6.1103 416923

[28] Guensch DP Fischer K Jung C Hurni S Winkler BM Jung B. Relationship between myocardial oxygenation and blood pressure: experimental validation using oxygenation-sensitive cardiovascular magnetic resonance. PLoS ONE. (2019) 14:e0210098. 10.1371/journal.pone.0210098 30650118 PMC6334913

[29] Fukuta H Little WC. The cardiac cycle and the physiologic basis of left ventricular contraction, ejection, relaxation, and filling. Heart Fail Clin. (2008) 4:1–11. 10.1016/j.hfc.2007.10.004 18313620 PMC2390899

[30] Brainin P Biering-Sørensen SR Møgelvang R de Knegt MC Olsen FJ Galatius S. Post-systolic shortening: normal values and association with validated echocardiographic and invasive measures of cardiac function. Int J Cardiovasc Imaging. (2019) 35:327–37. 10.1007/s10554-018-1474-2 30341672

[31] Voigt J-U Lindenmeier G Exner B Regenfus M Werner D Reulbach U. Incidence and characteristics of segmental postsystolic longitudinal shortening in normal, acutely ischemic, and scarred myocardium. J Am Soc Echocardiogr. (2003) 16:415–23. 10.1016/S0894-7317(03)00111-1 12724649

[32] Schuijf JD Shaw LJ Wijns W Lamb HJ Poldermans D de Roos A. Cardiac imaging in coronary artery disease: differing modalities. Heart. (2005) 91:1110–7. 10.1136/hrt.2005.061408 16020614 PMC1769025

[33] Westenberg JJM. CMR for assessment of diastolic function. Curr Cardiovasc Imaging Rep. (2011) 4:149–58. 10.1007/s12410-011-9070-z 21475412 PMC3047728

[34] Dongaonkar RM Stewart RH Geissler HJ Laine GA. Myocardial microvascular permeability, interstitial oedema, and compromised cardiac function. Cardiovasc Res. (2010) 87:331–9. 10.1093/cvr/cvq145 20472566 PMC2895547

[35] Guensch DP Yu J Nadeshalingam G Fischer K Shearer J Friedrich MG. Evidence for acute myocardial and skeletal muscle injury after serial transthoracic shocks in healthy swine. PLoS ONE. (2016) 11:e0162245. 10.1371/journal.pone.0162245 27611090 PMC5017707

[36] Schinkel AFL Bax JJ Geleijnse ML Boersma E Elhendy A Roelandt JRTC. Noninvasive evaluation of ischaemic heart disease: myocardial perfusion imaging or stress echocardiography? Eur Heart J. (2003) 24:789–800. 10.1016/S0195-668X(02)00634-6 12727146

[37] Leong-Poi H Rim S-J Le DE Fisher NG Wei K Kaul S. Perfusion versus function: the ischemic cascade in demand ischemia. Circulation. (2002) 105:987–92. 10.1161/hc0802.104326 11864930

[38] Gavara J Rodriguez-Palomares JF Valente F Monmeneu JV Lopez-Lereu MP Bonanad C. Prognostic value of strain by tissue tracking cardiac magnetic resonance after ST-segment elevation myocardial infarction. JACC Cardiovasc Imaging. (2018) 11:1448–57. 10.1016/j.jcmg.2017.09.017 29248649

[39] Eitel I Stiermaier T Lange T Rommel K-P Koschalka A Kowallick JT. Cardiac magnetic resonance myocardial feature tracking for optimized prediction of cardiovascular events following myocardial infarction. JACC Cardiovasc Imaging. (2018) 11:1433–44. 10.1016/j.jcmg.2017.11.034 29454776

[40] Fischer K Obrist SJ Erne SA Stark AW Marggraf M Kaneko K. Feature tracking myocardial strain incrementally improves prognostication in myocarditis beyond traditional CMR imaging features. JACC Cardiovasc Imaging. (2020) 13:1891–901. 10.1016/j.jcmg.2020.04.025 32682718

[41] Riffel JH Siry D Salatzki J Andre F Ochs M Weberling LD. Feasibility of fast cardiovascular magnetic resonance strain imaging in patients presenting with acute chest pain. PLoS ONE. (2021) 16:e0251040. 10.1371/journal.pone.0251040 33939756 PMC8092784

[42] Argyle RA Ray SG. Stress and strain: double trouble or useful tool? Eur J Echocardiogr. (2009) 10:716–22. 10.1093/ejechocard/jep066 19525297

[43] Schneeweis C Qiu J Schnackenburg B Berger A Kelle S Fleck E. Value of additional strain analysis with feature tracking in dobutamine stress cardiovascular magnetic resonance for detecting coronary artery disease. J Cardiovasc Magn Reson. (2014) 16:72. 10.1186/s12968-014-0072-2 25316531 PMC4180849

[44] Romano S Romer B Evans K Trybula M Shenoy C Kwong RY. Prognostic implications of blunted feature-tracking global longitudinal strain during vasodilator cardiovascular magnetic resonance stress imaging. JACC Cardiovasc Imaging. (2020) 13:58–65. 10.1016/j.jcmg.2019.03.002 31005520 PMC6745296

[45] Yimcharoen S Zhang S Kaolawanich Y Tanapibunpon P Krittayaphong R. Clinical assessment of adenosine stress and rest cardiac magnetic resonance T1 mapping for detecting ischemic and infarcted myocardium. Sci Rep. (2020) 10:14727. 10.1038/s41598-020-71722-3 32895408 PMC7477195

[46] Walcher T Manzke R Hombach V Rottbauer W Wöhrle J Bernhardt P. Myocardial perfusion reserve assessed by T2-prepared steady-state free precession blood oxygen level-dependent magnetic resonance imaging in comparison to fractional flow reserve. Circ Cardiovasc Imaging. (2012) 5:580–6. 10.1161/CIRCIMAGING.111.971507 22855554

[47] Ochs MM Kajzar I Salatzki J Ochs AT Riffel J Osman N. Hyperventilation/Breath-hold maneuver to detect myocardial ischemia by strain-encoded CMR: diagnostic accuracy of a needle-free stress protocol. JACC Cardiovasc Imaging. (2021) 14:1932–44. 10.1016/j.jcmg.2021.02.022 33865775

[48] Bucius P Erley J Tanacli R Zieschang V Giusca S Korosoglou G. Comparison of feature tracking, fast-SENC, and myocardial tagging for global and segmental left ventricular strain. ESC Heart Fail. (2019) 7:523–32. 10.1002/ehf2.12576 31800152 PMC7160507

[49] Fischer K Linder OL Erne SA Stark AW Obrist SJ Bernhard B. Reproducibility and its confounders of CMR feature tracking myocardial strain analysis in patients with suspected myocarditis. Eur Radiol. (2021). 10.1007/s00330-021-08416-5. [Epub ahead of print]. PMC9038796 34932165

[50] Fischer K Guensch DP Jung B King I Von Tengg-Kobligk H Giannetti N. Insights Into Myocardial Oxygenation and Cardiovascular Magnetic Resonance Tissue Biomarkers in Heart Failure With Preserved Ejection Fraction. Circ Heart Fail. (2022). 10.1161/CIRCHEARTFAILURE.121.008903. [Epub ahead of print]. 35038887

[51] Zhang Q Burrage MK Lukaschuk E Shanmuganathan M Popescu IA Nikolaidou C. Toward replacing late gadolinium enhancement with artificial intelligence virtual native enhancement for gadolinium-free cardiovascular magnetic resonance tissue characterization in hypertrophic cardiomyopathy. Circulation. (2021) 144:589–99. 10.1161/CIRCULATIONAHA.121.054432 34229451 PMC8378544

[52] Lakkireddy D Aronow WS Bateman T McGhee I Nair C Khan IA. Does beta blocker therapy affect the diagnostic accuracy of adenosine single-photon-emission computed tomographic myocardial perfusion imaging? Am J Ther. (2008) 15:19–23. 10.1097/MJT.0b013e31804c71a7 18223349

