# Supplementary material for: Combined Analysis of Myocardial Deformation and Oxygenation Detects Inducible Ischemia Unmasked by Breathing Maneuvers in Chronic Coronary Syndrome
Source: Front Cardiovasc Med. 2022 Feb 24;9:800720. doi: 10.3389/fcvm.2022.800720 (PMC8907543; doi:10.3389/fcvm.2022.800720)
Supplement: Supplementary file 1 [file Data_Sheet_1.PDF]

Combined analysis of myocardial deformation and oxygenation detects inducible ischemia unmasked by breathing maneuvers in chronic coronary syndrome

Barbara Spicher, MD[1 †]; Kady Fischer, PhD[1 †]; Zoe A. Zimmerli, MD[1]; Kyohei Yamaji, MD[2]; Yasushi Ueki, MD[2]; Carina N. Bertschinger[3]; Bernd Jung, PhD[3]; Tatsuhiko Otsuka, MD[2]; Marius Reto Bigler, MD[2]; Christoph Gräni, MD, PhD[2]; Hendrik von Tengg-Kobligk, MD[3]; Lorenz Räber, MD, PhD[2]; Balthasar Eberle, MD[1]; Dominik P. Guensch, MD[1,3*]

[1] Department of Anaesthesiology and Pain Medicine, Inselspital, University Hospital Bern, University of Bern, Bern, Switzerland
[2] Department of Cardiology, Inselspital, University Hospital Bern, University of Bern, Bern, Switzerland
[3] Department of Diagnostic, Interventional and Paediatric Radiology, Inselspital, University Hospital Bern, University of Bern, Bern, Switzerland
[†]Shared first author status

Supplemental Figure 1: Radial strain during the cardiac cycle 2

Supplemental Figure 2: Radial strain measurements 3

Supplemental Figure 3: Heart rate adjusted TTP 4

Supplemental Figure 4: Impact of medication on CMR responses 5

Supplemental Table 1: Association of FT-CMR between OS-cine images and the gold-standard cine stack 6

Supplemental Table 2: Regional CMR responses over the breathing maneuvers 7

Supplemental Table 3: Correlation to T1 and T2 Mapping 8

Supplemental Figure 1: Radial strain during the cardiac cycle

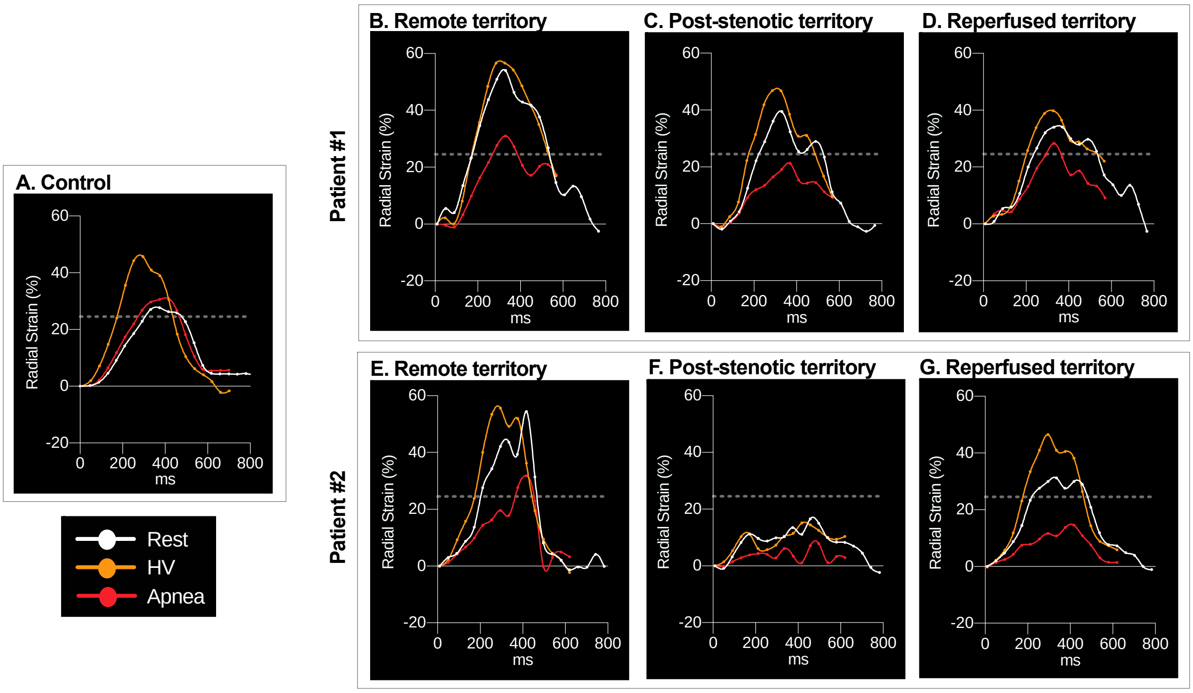

Radial strain during the cardiac cycle for the same three subjects described in Figure 2. The dotted line denotes the level of 1 SD below mean peak strain (PS) of control subjects at rest (24.5%). The same trends were observed with radial strain as described with circumferential strain. More positive PS values represent augmented systolic radial thickening.

Supplemental Figure 2: Radial strain measurements

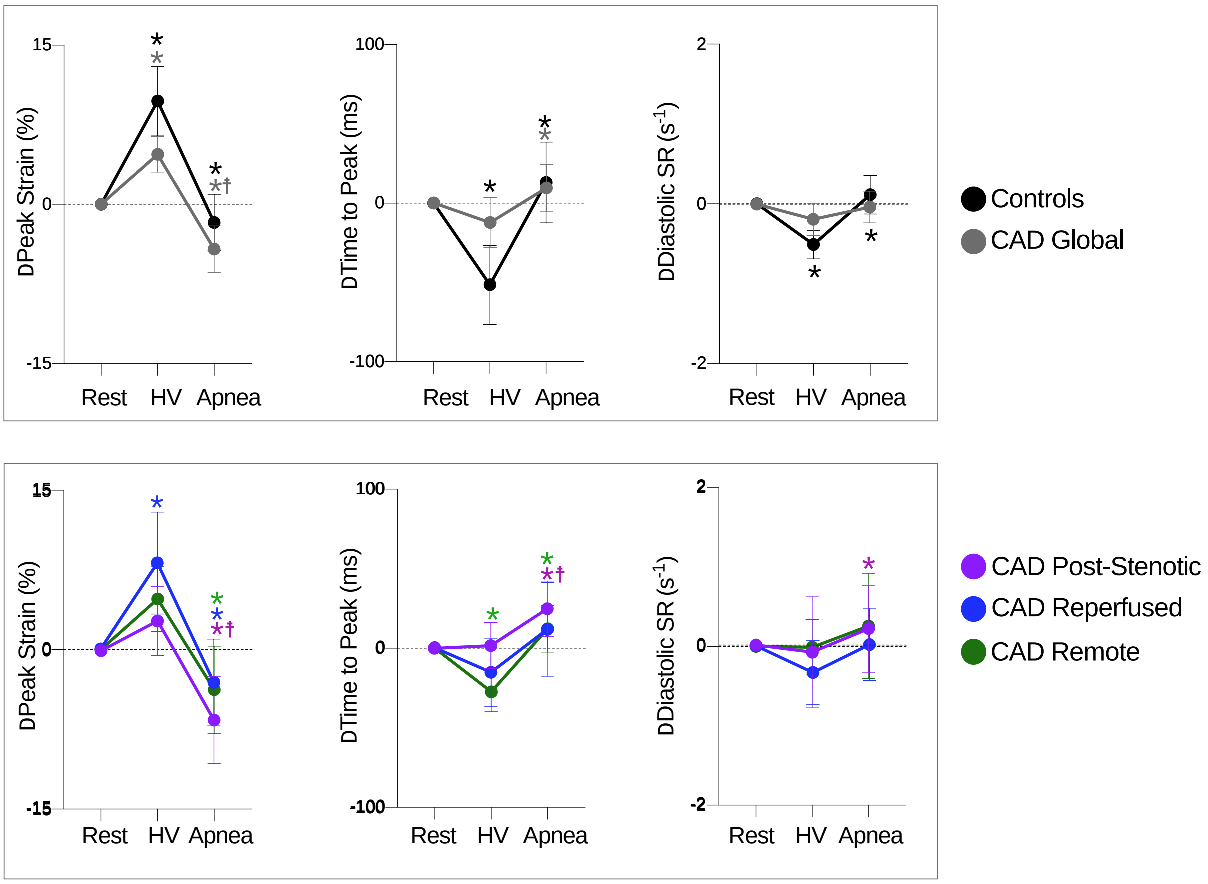

Data are mean±95% confidence intervals of measurements of global peak strain (PS), time to peak strain (TTP) and early diastolic strain rate (dSR) for healthy controls (black) and CAD patients (grey) in the radial orientation. Data for angiography defined regions are shown in the bottom row. *p<0.05 between consecutive steps, † p<0.05 at rest vs. apnea.

Supplemental Figure 3: Heart rate adjusted TTP

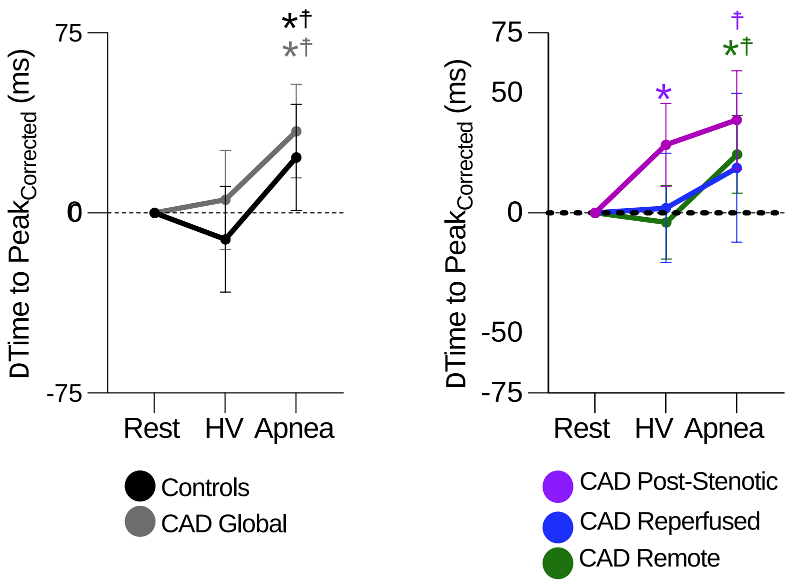

Mean±95% confidence intervals of circumferential time to peak strain (TTP) adjusted by the square root of the RR interval. *$p<0.05$ between consecutive steps, † $p<0.05$ at rest vs. apnea.

Supplemental Figure 4: Impact of medication on CMR responses

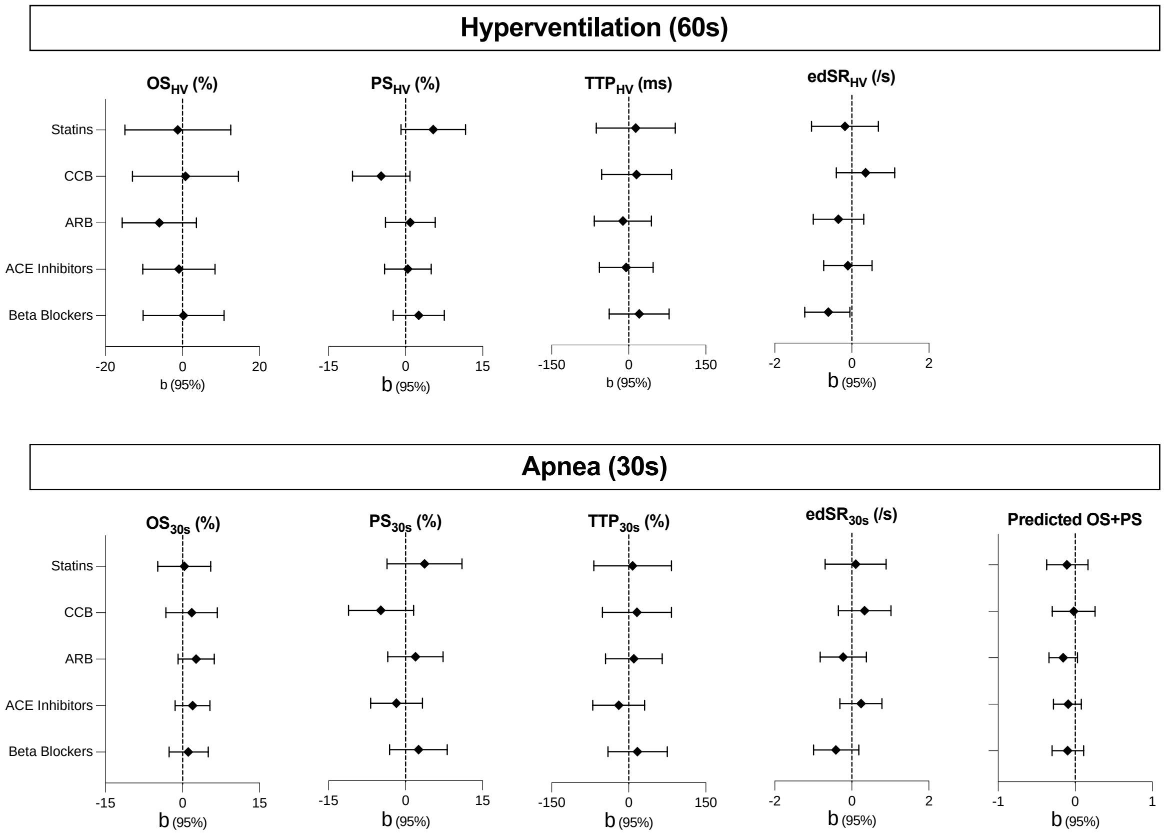

Beta parameter coefficients and 95% confidence intervals are shown for the association of medication and the post-stenotic CMR results during the breathing maneuver determined by linear regression. The bottom-left depicts the predicted probability variable derived from the logistic regression analysis combining the oxygenation-sensitive (OS) and peak strain (PS) response at 30s into apnea. There was no statistically significant association between medication and CMR results measured during hyperventilation (HV) or apnea (30s). ACE: angiotensin-converting enzyme, ARB: angiotensin receptor blocker, CCB: calcium channel blocker, edSR: early diastolic strain rate, TTP: time to peak strain.

Supplemental Table 1: Association of FT-CMR between OS-cine images and the gold-standard cine stack

Parameter	Circumferential ICC	Circumferential p	Radial ICC	Radial p
**Peak Strain**				
Global	0.900	<0.001	0.909	<0.001
Post-stenotic	0.801	<0.001	0.848	<0.001
Reperfused	0.814	0.001	0.928	<0.001
Remote	0.905	<0.001	0.915	<0.001
**Time to Peak Strain**				
Global	0.674	0.008	0.689	0.006
Post-stenotic	0.808	<0.001	0.828	<0.001
Reperfused	0.810	<0.001	0.884	<0.001
Remote	0.844	<0.001	0.859	<0.001
**Diastolic Strain Rate**				
Global	0.751	<0.001	0.888	<0.001
Post-stenotic	0.775	<0.001	0.487	0.094
Reperfused	0.715	0.005	0.889	<0.001
Remote	0.888	<0.001	0.743	0.002

Intraclass correlation coefficients (ICC) are shown for the comparison of strain parameters from the OS-cine versus the standard short-axis ECG-gated cine stack, for both the global myocardium, and individual territories (n=25).

Supplemental Table 2: Regional CMR responses over the breathing maneuvers

	Rest	HV	Apnea	P value (Rest vs. HV)	P value (HV vs. Apnea)	P value (Rest vs. Apnea)
**CIRCUMFERENTIAL STRAIN**						
**CAD Post-Stenotic Territory**						
PS (%)	-22.5±4.8	-22.8±5.2	-20.7 ±5.8	0.839	**0.004***	**0.017***
TTP (ms)	319±46	324±59	343±58	0.837	0.084	**0.022***
dSR (s^{-1})	1.72±0.80	1.56±0.65	1.50±0.60	0.911	0.998	0.953
**CAD Reperfused Territory**						
PS (%)	-18.7±5.6	-21.4±4.8	-17.9±4.4	**0.009***	**<0.001***	0.639
TTP (ms)	340±77	323±71	345±77	0.269	0.122	0.900
dSR (s^{-1})	1.57±0.99	1.48±0.70	1.37±0.56	0.996	0.876	0.975
**CAD Remote Territory**						
PS (%)	-21.3±4.5	-22.1±5.0	-20.4±4.1	0.312	**0.019***	0.315
TTP (ms)	337±43	314±41	348±77	**0.008***	**<0.001***	0.295
dSR (s^{-1})	1.84±0.88	1.61±0.56	1.69±0.76	0.645	0.910	0.754
**RADIAL STRAIN**						
**CAD Post-Stenotic Territory**						
PS (%)	45.5±16.4	48.3±18.3	39.0±15.6	0.194	**0.002***	**0.008***
TTP (ms)	318±46	320±57	343±58	0.971	**0.014***	**0.008***
dSR (s^{-1})	-3.57±1.74	-3.75±1.86	-3.31±1.63	0.754	**0.049***	0.590
**CAD Reperfused Territory**						
PS (%)	34.4±14.8	43.6±16.2	31.2±12.5	**<0.001***	**<0.001***	0.280
TTP (ms)	337±76	322±70	349±81	0.386	0.594	0.066
dSR (s^{-1})	-2.65±-1.54	-3.02±1.78	-2.66±1.45	0.124	0.375	0.999
**CAD Remote Territory**						
PS (%)	42.1±13.9	46.1±14.0	38.3±11.7	0.070	**<0.001***	0.101
TTP (ms)	337±76	306±42	348±53	**<0.001***	**<0.001***	0.169
dSR (s^{-1})	-3.47±1.41	-3.51±1.17	-3.22±1.76	0.975	0.600	0.713
**OXYGENATION-SENSITIVE RESPONSE (%)**						
Post-Stenotic		-5.5±10.5	1.6±3.9	**0.005***	**0.011***	**<0.001***
Reperfused		-3.3±8.4	1.3±6.7	**0.049***	0.367	**0.041***
Remote		-7.1±11.3	4.9±5.7	**<0.001***	**<0.001***	0.059

Mean±SD radial peak strain (PS), time to peak strain (TTP) and early diastolic strain rate (dSR) are shown for rest, post hyperventilation (HV) and at the 30s time-point in apnea (Apnea), along with the regional myocardial oxygenation-sensitive (OS) percent responses for hyperventilation and apnea. *p<0.05 from the post-hoc analysis from the mixed-effects models.

Supplemental Table 3: Correlation to T1 and T2 Mapping

		Baseline T1	Baseline T2	After Apnea T1	After Apnea T2
Global	PS (%)	0.13	0.12	0.23	0.04
Global	TTP (ms)	0.05	0.14	-0.21	-0.09
Global	dSR (/s)	-0.18	-0.36†	-0.25	-0.14
Global	OS (%)			-0.45*	0.01
Post-Stenotic	PS (%)	0.48*	0.18	0.35†	-0.01
Post-Stenotic	TTP (ms)	-0.28	-0.08	-0.15	-0.07
Post-Stenotic	dSR (/s)	-0.07	-0.20	-0.19	-0.01
Post-Stenotic	OS (%)			-0.26	0.15

Pearson's correlation coefficients are shown for the association of circumferential feature tracking and myocardial oxygenation measurements at rest and after 30s of apnea in comparison to T1 and T2 maps. *p<0.05 (green), 0.05≤p<0.10 (blue).
dSR: early diastolic strain rate, OS: oxygenation-sensitive, PS: peak strain, TTP: time to peak strain.
